# Enhancing thermostability of Moloney murine leukemia virus reverse transcriptase through greedy combination of multiple mutant residues

**DOI:** 10.1186/s40643-025-00845-0

**Published:** 2025-02-20

**Authors:** Youhui Yang, Jie Zhang, Zhong Li, Hao Qi

**Affiliations:** 1 School of Chemical Engineering and Technology, Tianjin University, Tianjin, China; 2 Key Laboratory of Systems Bioengineering (Ministry of Education), Tianjin University, Tianjin, China

**Keywords:** Reverse transcriptase, Protein engineering, Thermostability, Melting temperature

## Abstract

Reverse transcription is crucial in bioengineering and biomedical fields, particularly for genome sequencing and virus diagnosis. Enhancing the thermostability of reverse transcriptase can significantly improve its efficiency and accuracy by enabling it to function at higher temperatures, thereby reducing RNA secondary structures and minimizing interference from contaminating enzymes, particularly in clinical samples. Here, using a combinatorial strategy, a variant of Moloney Murine Leukemia Virus reverse transcriptase (MMLV RT) with improved activity across a wide temperature range (30–50 °C) was identified and maintained 100% activity after incubation at 50 °C for 10 min. Eleven hot-spot residues were mutated in various combinations, and the mutant proteins were rapidly expressed in a cell-free system for reverse transcription activity testing. Variant M5, which carries five mutated residues (E47K/E280R/T284R/L413G/D631V), exhibited enhanced thermostability and activity compared to any variant with a single residue mutation. Using purified recombinant protein for precise characterization, the melting temperature (Tm) of M5 increased by 4.7 °C when assembled with a nucleotide template-primer (T/P). Consequently, the half-life of M5 at 50 °C extended to approximately 60 min, in contrast to less than 4 min for the wild type. These findings demonstrate that the epistasis of combining multiple mutant residues holds excellent potential for significantly enhancing enzyme activity, even with existing knowledge. This heat-stable MMLV RT variant M5 may potentially improve efficiency and accuracy in molecular biology research and clinical diagnostics.

**Supplementary Information:**

The online version contains supplementary material available at 10.1186/s40643-025-00845-0.

## Introduction

Reverse transcriptases (RTs), an enzyme present in retroviruses, exhibits both RNA- and DNA-dependent DNA polymerase, as well as RNase H activities (Temin 1970; Prabaharan et al. 2024). Moloney Murine Leukemia Virus reverse transcriptase (MMLV RT) is widely utilized in cDNA synthesis due to its superior catalytic activity and fidelity (Yasukawa et al. 2010). As a result, it is extensively applied in various techniques, including RT-PCR, cDNA cloning and library construction, rapid amplification of cDNA ends, and RNA sequencing (Arezi et al. 2009; Martín-Alonso et al. 2021). Typically, MMLV RT is recommended for use at 37 °C; however, the secondary structure of RNA at this temperature adversely impacts nucleotide primer binding and the overall reverse transcription process. Consequently, it results in truncated cDNA production or complete cDNA transcription failure (Harrison et al. 1998). Elevating the reaction temperature to around 45 °C can mitigate these issues by destabilizing RNA secondary structures and enhancing transcription efficiency (Arezi and Hogrefe 2009). Consequently, improving the thermostability of reverse transcriptase is vital for optimizing reverse transcription reactions.

During the COVID-19 pandemic, MMLV RT became one of the most widely utilized enzymes in SARS-CoV-2 diagnostics worldwide due to its critical role in reverse transcription-PCR (RT-PCR) assays (Cerda et al. 2024). Enhancing the enzyme’s thermostability is essential for clinical diagnostics. It enables the enzyme to function accurately and effectively at higher temperatures by reducing the RNA secondary structures and minimizing the interference from contaminating enzymes, which is especially crucial for clinical samples. Given its significance in diagnostics and bioengineering, efforts to enhance MMLV RT’s activity and thermostability have been pursued. The pentuple mutant (E69K/E302R/W313F/L435G/N454K) was generated through a combination of random and saturation mutagenesis, which exhibited high thermal stability at 55 °C incubating without T/P (Arezi and Hogrefe 2009). However, the determination of the reaction rates may be inaccurate due to the MMLV RT RNA polymerase’s reaction speed of up to 327 nM/s (Palikša et al. 2018). Furthermore, stabilization methods have been applied through site-directed mutagenesis. The M4 variant (E286R/E302K/L435R/D524A), also obtained via site-directed mutagenesis, its activity decreased to 50% after incubation at 50 °C (Yasukawa et al. 2010). By contrast, the quadruple mutant (D108R/E286R/V433R/D524A) was obtained by introducing charged residues into continuous surface hydrophobic residues, showing high thermal stability. However, its activity decreased to 70% after incubation at 50 °C for 10 min (Konishi et al. 2014). Of course, it is important to note that in addition to the aforementioned methods, other strategies can be employed to enhance the thermal stability of MMLV RT. The double mutant (A551C/T662C), generated by introducing a disulfide bond through site-directed mutagenesis, exhibited enhanced thermal stability of reverse transcriptase, with an observed reduction in activity to 17% after treatment at 50 °C for 3 min (Narukawa et al. 2021). Despite the isolation of several thermostable variants, their thermostability remains inadequate. Consequently, enhancing the thermal stability of RTs continues to be a significant research objective.

In protein engineering, epistasis refers to the interactions between multiple mutant residues that yield effects beyond their individual contributions. For example, a variant of MMLV RT carrying four mutations (E286R/E302K/L435R/D524A) has been reported to exhibit enhanced activity around 60 °C (Yasukawa et al. 2010). This case underscores the significance of leveraging existing knowledge of mutations to strategically combine them, thereby exploiting these interactions to enhance protein activity beyond the sum of their isolated effects. By capitalizing on the known impacts of mutations, this approach has significant potential to substantially improve enzyme design, including enhanced catalytic activity, stability, and specificity. Thus, utilizing epistasis based on existing knowledge can lead to significant performance gains in engineered proteins across various applications.

In this study, we demonstrate an enhanced thermostability and activity of MMLV RT through a rapid combinatorial experiment targeting key hot-spot residues. We evaluated 11 mutant residues previously identified as critical for reverse transcription, either by eliminating RNase H activity (Divbandi et al. 2024) or by improving template-primer (T/P) binding affinity (Arezi and Hogrefe 2009). We identified a new variant, M5, which includes five specific mutations (E47K/E280R/T284R/L413G/D631V) by testing various combinations of these residues. This variant significantly enhances thermostability compared to any individual mutant residue. This unique combination of mutations in M5 is distinct from previously reported variants and exhibits high reverse transcription activity across a temperature range of 30–50 °C. Notably, its activity remained at 100% even after incubation at 50 °C for 10 min. Furthermore, both the half-life of enzymatic activity and the melting temperature (Tm) were carefully measured, revealing substantial improvements respectively.

## Materials and methods

### Plasmid construction

The Moloney murine leukemia virus reverse transcriptase (MMLV RT) (GenBank accession number: J02255) and Twin-Strep-tag (GenBank accession number: MN882188.1) were as codon optimized for *E. coli* and synthesized by BGI Tech Solutions (Beijing, Liuhe) Co., Ltd., and the MMLV RT and Twin-Strep-tag were cloned into the vector pET23a backbone yielding the pET23a-MMLV-Twin-strep using a ClonExpress MultiS One Step Cloning Kit (Vazyme, C113-01). The *mlv* gene was amplified with PrimeSTAR Max DNA Polymerase (Takara, R045) using the specific primers (Supplementary Table S1) and further inserted into the pET28a vectors containing an N-terminal His_6_-tag using a ClonExpress II One Step Cloning Kit (Vazyme, C112-01). The recombinant product was transformed into *E.coli* XL10-Gold Competent Cell.

### Mutation of reverse transcriptase

Using pET23a-MMLV-Twin-strep as templates, gene splicing by overlap extension PCR (SOE PCR) was used to insert mutations in specific places of the MMLV sequence. The UFB1 and URB1 primers (Supplementary Table S1) were altered at the 5’ end by a desthiobiotin moiety on a PEG linker to aid in PCR product purification without agarose gel electrophoresis. To prepare individual DNA fragments, these tagged primers were utilized in conjunction with the relevant mutagenic primers mentioned below (Supplementary Table S1). The PrimeSTAR Max DNA Polymerase (Takara, R045) is used in all PCR amplification reactions. A volume of 5 μL of BeaverBeads^®^ Streptavidin (BEAVER, 22308-100) was combined with 50 μL PCR reactions that had been diluted with 145 μL binding buffer (10 mM Tris-HCl pH 7.5, 0.05% Tween-20, 1 mM EDTA, and 1 M NaCl) to purify DNA fragments. After incubation on a rocking platform at 4 °C for 30 min, the suspension was centrifuged (1,000 g, 1 min), and the supernatant was discarded. The streptavidin beads were washed three times with 200 μL of binding buffer, followed by incubating for 30 min at 55 °C in 50 μL of elution solution (2.5 mM biotin, 5 mM Tris-HCl pH 8.0, 0.5 mM EDTA, and 0.05% Tween-20) for recombination of DNA fragments into the full-length MMLV sequence by SOE PCR with standard UF2 and UR2 primers. Thermocycling was done for 2 min at 95 °C, 30 cycles of 30 s at 95 °C, 30 s at 55 °C, and 40 s at 72 °C, and then a 5 min extension at 72 °C. The band size was verified by electrophoresis and Sanger sequencing for the PCR products, which were then refrigerated at -20 °C before use.

### Cell-free expression of reverse transcriptase and in vitro reverse transcription

The S30 cell extract was prepared as described in our previous study (Wu et al. 2022). The standard Cell-Free Protein Synthesis system (CFPS) reaction was carried out in a tube to a final volume of 20 μL at 30 °C for 3 h. Each reaction contained the following components: 35% (V/V) cell extract, 40 mM HEPES, 130 mM potassium glutamate, 2 mM DTT, 18 mM magnesium acetate, 1 mM putrescine, 1.5 mM spermidine, 0.34 mM NAD, 0.5 mM of each CTP, GTP, and UTP, 0.3 mM CoA, 170 μg/mL tRNA, 34 μg/mL folinic acid calcium, 33.33 mM PEP, 4 μL of the PCR products and the remainder water. After the reaction, 20 μL of BeaverBeads™ Magrose Strep-Tactin (BEAVER, 70808-250) were mixed with 20 μL CFPS reactions that had been diluted with 160 μL binding buffer (10 mM Tris-HCl pH 8.0, 1 mM EDTA, and 150 mM NaCl) to purify MMLV RT. The tube was incubated at 4 °C for 30 min on a rocking platform. After centrifugation, the supernatant was discarded, and the beads were washed three times with 200 μL of binding buffer. The 60 μL of elution buffer (2.5 mM biotin, 10 mM Tris-HCl pH 8.0, 1 mM EDTA, 150 mM NaCl, and 0.05% Tween-20) were added to a tube on a rocking platform at 4 °C for 30 min.

A two-step RT-PCR assay was used to determine the efficiency of the reverse transcription reaction at different temperatures. The reverse transcription reaction is performed according to the following steps: the reaction mixture was prepared by 5 × MMLV buffer (250 mM Tris-HCl pH 8.3, 375 mM KCl, 15 mM MgCl_2_, and 50 mM DTT), 2.5 mM dNTP, 2 μM MSR-R4 primer, and 2 μL the corresponding RT. The reactions were incubated for three min at either 37–50 °C according to previous studies (Baba et al. 2017; Katano et al. 2017), followed by incubating on ice for 30 min. The MS2 RNA (Merck, 10165948001) (50 ng) was added, followed by incubating for 45 min at 37 °C or 50 °C, and then stopped when the mixtures were heated at 85 °C for 10 min. The cDNA synthesis yields were measured using QuantStudio 6. The reaction mixture for qPCR (20 μL) was prepared by mixing the reaction product of cDNA synthesis (2 μL), water (13.1 μL), 10 × PCR buffer (2 μL), 2.5 mM dNTP (1.6 μL), 10 μM G-F primer (0.4 μL) and 10 μM G-R primer (0.4 μL), 5 U/μL easytaq DNA polymerase (0.4 μL), and 4 × SYBR Green I (1 μL). The thermocycling conditions were as follows: 5 min at 94 °C, 40 cycles of 30 s at 94 °C, 30 s at 55 °C, and 20 s at 72 °C. During the amplification process, fluorescence signals were measured at the extension step.

The data were analyzed using the QuantStudio Real-Time PCR software version 1.2 from Applied Biosystems.

Quantitative analysis of the reverse transcription efficiency was evaluated according to previous studies (Matamoros et al. 2013). The threshold cycle (Ct) at which the fluorescence significantly exceeds the background was determined by the quantification software. The ΔCt value for each sample was calculated by subtracting the Ct value of the reference gene from the Ct value of the target gene. Subsequently, the relative yields were determined by calculating the 2^ΔCt^ value for each cDNA.

### Purification of recombinant proteins

The pET28a-MMLV-Twin-strep plasmid, which carries the nucleotide sequence encoding the MMLV RT with an N-terminal His_6_-tag, was used to transform the *E. coli* strain BL21(DE3) [*F − ompT hsdSB* (*rB−*, *mB−*) *gal dcm rne* 131(DE3)]. After being cultivated overnight, 5 mL of cells were added to 500 mL of kanamycin (50 μg/mL) Luria-Bertani broth and shaken to incubate at 37 °C. To promote protein expression, 0.5 mL of 1 M IPTG was added when the OD_600_ reached 0.6, and growth was continued at 18 °C for 16 h (Nuryana et al. 2022). After centrifugation at 4,000 rpm for 30 min, the pellets were suspended with 10 mL of lysis buffer containing 0.2 mg/mL lysozyme, 50 mM HEPES-KOH pH 7.6, 1 M NH_4_Cl, 10 mM MgCl_2_, 20 mM imidazole, 7 mM BME and 0.2% V/V Triton X-100 and incubated at 4 °C with shaking. The sample was sonicated for 10 min using the following settings: 190 W power output, 2 s on/8 s off pulses, ice-cold water bath but no floating ice. After centrifugation at 12,000 rpm for 30 min, the supernatant was collected and filtered. The subsequent purification was performed on the protein purification system (GE Healthcare) with a Ni-Sepharose prepacked column (GE Healthcare, HisTrap HP 5 mL) according to the manufacturer’s protocol using buffer A (50 mM HEPES-KOH pH 7.6, 1 M NH_4_Cl, 10 mM MgCl_2_, 20 mM imidazole, 7 mM BME) and buffer B (50 mM HEPES-KOH pH 7.6, 100 mM KCl, 10 mM MgCl_2_, 400 mM imidazole, 7 mM BME). The protein sample was collected according to the absorbance at 280 nm and was carried out desalted using prepacked PD-10 gel filtration columns (GE Healthcare, HiTrap Desalting 5 mL) to the storage buffer (50 mM HEPES-KOH pH7.6, 75 mM KCl, 3 mM MgCl_2_, 30% glycerol, 10 mM DTT) and stored at -80 °C before using. The protein concentration was determined using the Pierce™ 660 nm Protein Assay kit (Thermo Fisher, 22660) using bovine serum albumin as standard.

### Optimal temperature for reverse transcription

The EnzChek Reverse Transcriptase Assay Kit (Thermo Fisher Scientific, E22064) was used to determine the optimal temperature for MMLV RT. The kit primarily exploits the preferential selectivity of the PicoGreen reagent when associated with double-stranded DNA or RNA-DNA heteroduplexes compared to single-stranded nucleic acids or free nucleotides. The detection of the optimal temperature of MMLV RT was prepared as follows: the poly (A) ribonucleotide template (0.3 mg/mL template in 100 mM Tris-HCl, 0.5 mM EDTA, pH 8.1) and the oligo (dT)_16_ primer (5 μg/mL primer in 100 mM Tris-HCl, 0.5 mM EDTA, pH 8.1) were mixed and incubated at room temperature for 1 h. RTs (5 nM) were incubated in the presence or absence of T/P (poly(rA)-oligo(dT)_16_) for 3 min at a temperature range of 30 °C to 60 °C, followed by the addition of polymerization buffer (60 mM Tris-HCl, 60 mM KCl, 8 mM MgCl_2_, 13 mM DTT, 100 μM dTTP, pH 8.1), and incubated at a temperature range of 30 °C to 60 °C for 1 h. Added 2 μL of 200 mM EDTA to each reaction to stop the reaction. To each tube (27 μL), PicoGreen reagent (173 μL) was added, followed by mixing and incubation for 5 min at room temperature in the absence of light. We measured the fluorescence at 520 nm using a SPARK multimode microplate reader (TECAN, Waltham, MA) with an excitation wavelength of 480 nm.

### Half-lives determination

The Half-lives of the mutants were determined using the EnzChek Reverse Transcriptase Assay Kit (Thermo Fisher Scientific, E22064) according to the manufacturer′s protocols. The 5 nM enzymes were incubated at 50 °C in the presence or absence of T/P (poly(rA)-oligo(dT)_16_) for different times, followed by the addition of polymerization buffer (60 mM Tris-HCl, 60 mM KCl, 8 mM MgCl_2_, 13 mM DTT, 100 μM dTTP, pH 8.1), which was subsequently added and incubated at 37 °C for 1 h. To stop the reaction, add 2 μL of 200 mM EDTA to each reaction. To each tube (27 μL), PicoGreen reagent (173 μL) was added, followed by mixing, and incubated for 5 min at room temperature in the absence of light. The standard quantitative curve of fluorescence could represent the enzyme activity. The fluorescence was measured using a SPARK multimode microplate reader (TECAN, Waltham, MA) with excitation at 480 nm and emission at 520 nm.

### Thermal shift assay

Protein thermal shift assay (PTSA) was performed using a Protein Thermal Shift™ kit (Thermo Fisher Scientific, 4461146). It includes Protein Thermal Shift™ Dye, which is used to track the temperature ramping-induced denature/unfolding of various proteins. As the temperature rises, the protein unfolds and exposes its hydrophobic residues or surface, allowing the fluorescent dye to bind to the hydrophobic area and become unquenched. The Tm was determined as the midpoint of the melting curve. In brief, the reactions were set up in a tube to a final volume of 20 μL. To each well, 1 μg of purified recombinant protein was added with 5 μL of thermal stability buffer and 2.5 μL of 8 × dye with 100 ng T/P (this concentration is expressed based on RNA). The temperature gradient was set in the range of 30˚C to 95˚C with a ramp rate of 0.05˚C/s, and the fluorescence was measured with excitation at 520 nm and emission at 558 nm. The Tm was calculated using Protein Thermal Shift Software 1x (Applied Biosystems). For each set, samples were made in quadruple, and at least three biologically independent experiments were conducted.

### Molecular dynamics simulations

The predicted structures of WT was prepared using AlphaFold2, and the predicted structure of the M5 variant was then obtained by point mutation of amino acids at the target site using Chimera. The molecular dynamics simulations were performed using Gromacs 2022.3. The Gromos 54a7 force field was utilized, with the Tip3p water model serving as the solvent under static conditions of 300 K temperature and 1 bar pressure (Sinha et al. 2023). To neutralize the system’s total charge, Na^+^ and Cl^−^ ions were introduced. The simulation system employed the steepest descent method for energy minimization, followed by isothermal-isobaric (NPT) equilibrium for 100,000 steps, each with a coupling constant of 0.1 ps and a duration of 100 ps. A free molecular dynamics simulation was then conducted over 500,000 steps, corresponding to a total duration of 50 ns, with a step length of 1 fs. Following the simulation, the root-mean-square variance (RMSD), root-mean-square fluctuation (RMSF) and radius of gyration (Rg) of the amino acid motion locus were analyzed using the built-in tool of the software in order to evaluate the stability of the protein WT and mutant protein M5.

### Fidelity assay

The fidelity of the WT and M5 variant were determined using the next-generation sequencing (NGS) described previously (Narukawa et al. 2021; Okano et al. 2018b; Yasukawa et al. 2017). Briefly, the cDNA synthesis reactions (20 μL) were performed with 34.4 ng/μL RTs in 1 × MMLV buffer (50 mM Tris-HCl pH 8.3, 75 mM KCl, 3 mM MgCl_2_, and 10 mM DTT), 0.125 mM dNTP, 0.1 μM MSR-R4 primer, and 1 μL 50 ng/μL MS2 RNA. The reactions were incubated for 45 min at 37 °C and then at 85 °C for 10 min. Next, the reaction mixture for PCR (50 μL) was prepared with TransStart^®^*FastPfu* DNA Polymerase (TransGen Biotech, AP221-01) in 1 × TransStart^®^*FastPfu* buffer, 0.2 μM MSQ-F3, 0.2 μM MSQ-R3, 0.2 mM dNTP, 2.5 U TransStart^®^*FastPfu* DNA Polymerase and 5 μL cDNA. The PCR reaction was performed for 2 min at 95 °C, 25 cycles of 20 s at 95 °C, 20 s at 55 °C, and 10 s at 72 °C followed by a 5 min extension at 72 °C. The PCR products were analyzed with 1.5% agarose gel to confirm the size of the band and subsequently purified using SPARKeasy Gel DNA Extraction Kit (Sparkjade, AE0101-B). The library was prepared using a PCR-free method and NGS sequencing was performed by Azenta Life Sciences.

## Results

### Epistasis of combinatorial hot-spot residues in MMLV reverse transcriptase

**Fig. 1:**
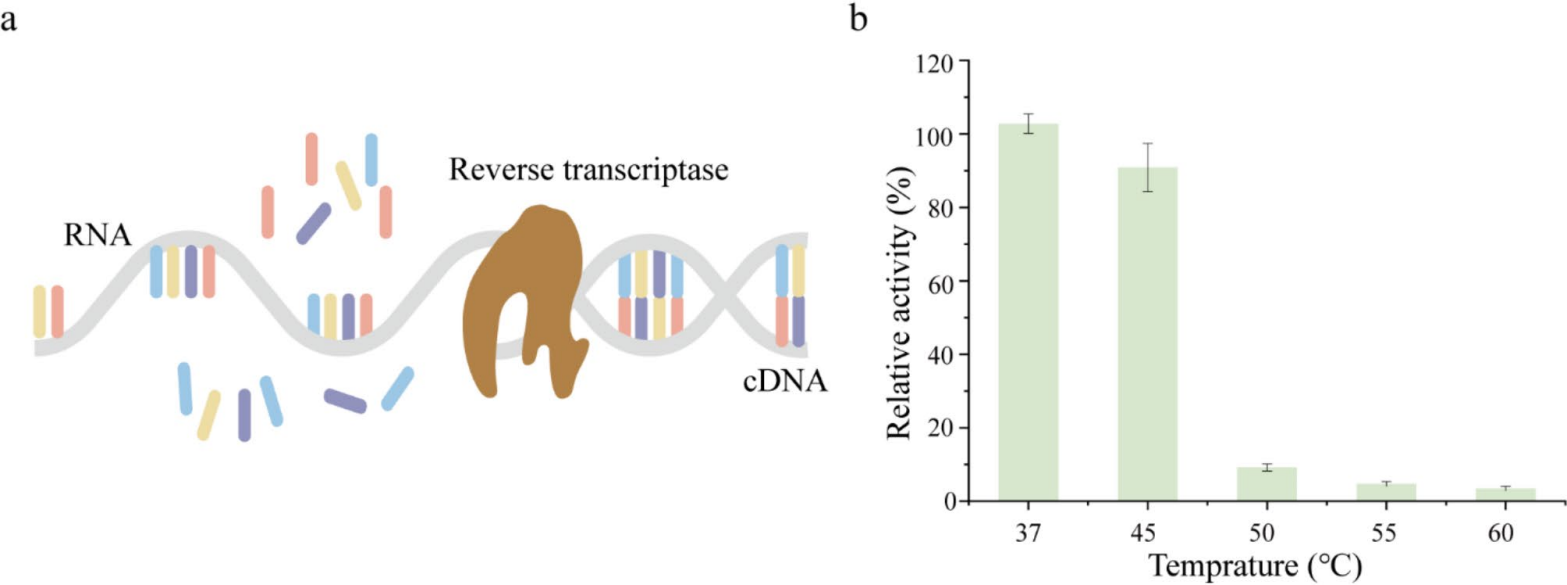
Process and thermal stability of MMLV RT. **a** Reverse transcription process by reverse transcriptase. **b** The effects of thermal treatment on the RNA-dependent DNA polymerase activities of MMLV RT were investigated. Relative activity is defined as the ratio of the activity at 37 °C. The values in the graph are the mean values of three independent experiments, and the error line represents the standard deviation

Reverse transcription is the process by which single-stranded DNA is synthesized from a single-stranded RNA template, and the synthesis is facilitated by the RT, with the four ribonucleotides serving as substrates (Fig. 1a). Due to their high catalytic activity and fidelity, MMLV RT is the most extensively utilized in conventional cDNA synthesis (Kimmel et al. 1987). This wild-type (WT) enzyme operates optimally at approximately 37 °C. The purified recombinant WT was evaluated across a range of temperatures. At the optimal temperature of 37 °C, the enzyme demonstrated maximal activity while minimizing the risks of denaturation and functional loss. However, as temperatures increased, a significant decline in reverse transcriptase activity was observed, with nearly complete loss of function at 50 °C (Fig. 1b). This finding aligns with previous reports, which showed a 50% reduction in the enzyme’s initial activity after a 10 min incubation at 44 °C (Yasukawa et al. 2008). This limitation underscores the necessity of enhancing the enzyme’s thermostability, which has become a crucial research focus. Proposed strategies to improve MMLV RT stability include eliminating RNase H activity (Gerard et al. 2002) and enhancing T/P binding affinity (Arezi and Hogrefe 2009; Matamoros et al. 2013). This underscores the importance of maintaining the enzyme within its optimal temperature range to ensure effective performance in related applications.

**Fig. 2:**
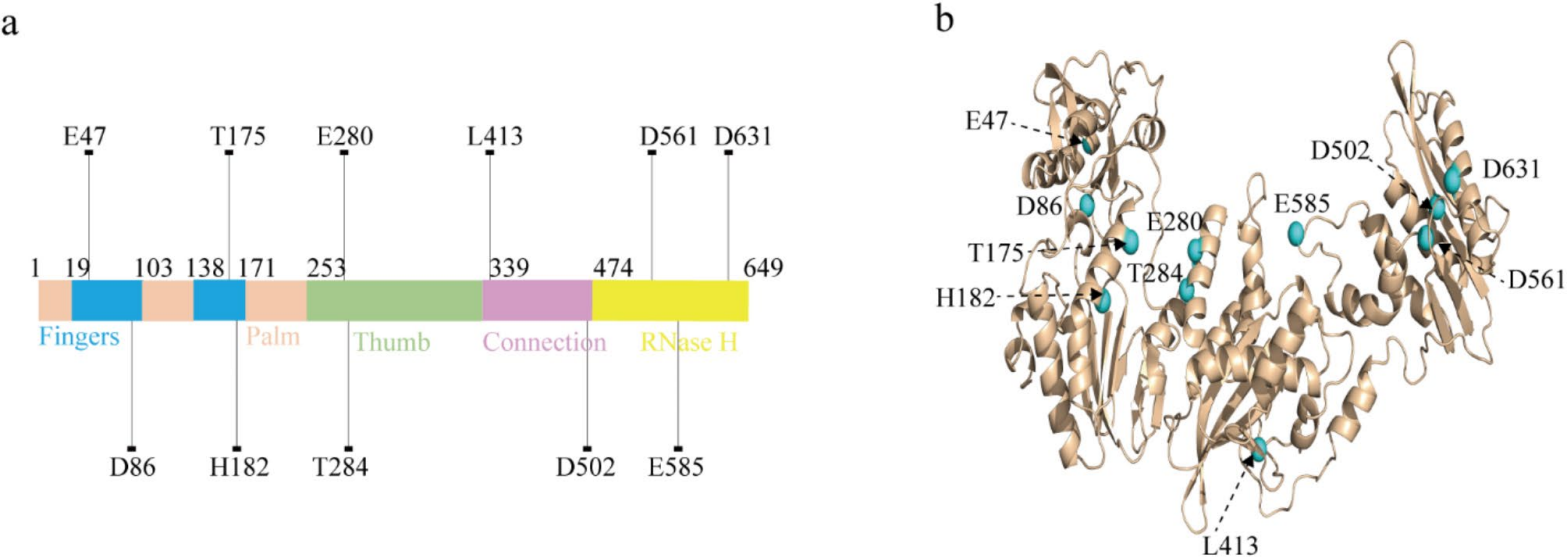
Hot-spot residues in MMLV RT. **a** Distribution of 11 residues on MMLV RT structure. **b** The cartoon representation of the crystal structure of MMLV RT (PDB: 5DMQ) with 11 residues, which were highlighted as aquamarine spheres

MMLV RT possesses three essential enzymatic activities: DNA-dependent DNA polymerase, RNase H, and RNA-dependent DNA polymerase, the latter of which is responsible for synthesizing cDNA from an RNA template. During reverse transcription, MMLV RT first binds to the T/P complex, then employs its RNA-dependent DNA polymerase activity to synthesize cDNA. In MMLV RT, the crystal structure of the fingers, palm, thumb, connection, and RNase H were determined based on the structure of the full-length MMLV RT (PDB: 5DMQ) (Tang et al. 2016). Building upon these functional characteristics, we identified 11 hot-spot residues on MMLV RT for modification. These residues are widely distributed in different functional regions of MMLV RT (Fig. 2a) and are also reported in the literature. (i) Four hydrophobic residues (E47, D86, L413, and E585) located on the molecular surface were selected to increase the surface charge (Fig. 2b) (Arezi and Hogrefe 2009; Baranauskas et al. 2012; Konishi et al. 2014). (ii) Two hydrophobic or polar residues (T175 and H182) located around the activity center of the enzyme were selected to stabilize the hydrophobic core (Das et al. 2004). (iii) Two residues (E280 and T284) are located in the binding pocket of the substrate to increase the binding force with the T/P (Nowak et al. 2013). (iv) Three residues (D502, D561, D631) located around the activity center of the RNase H were selected to eliminate the activity of RNase H (Lim et al. 2006).

**Fig. 3:**
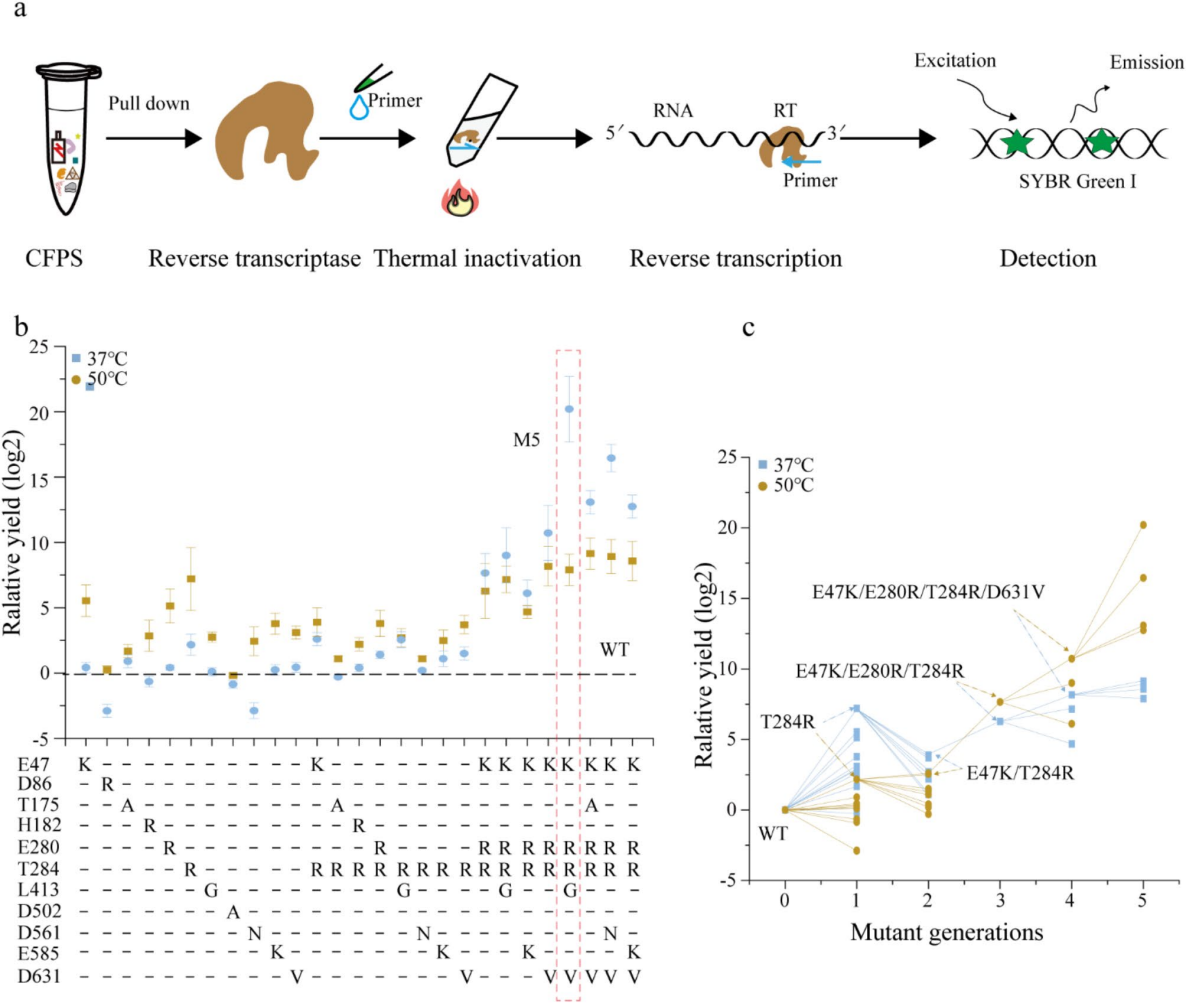
Fast testing of mutant MMLV RT. **a** The workflow shows the process of reverse transcriptase screening. RTs was expressed in the cell-free protein expression system and purified by magnetic beads, which were incubated with the primer at 37–50 °C, and then RNA was added for reverse transcription reaction. The PCR products were detected by adding a fluorescent dye capable of intercalating in double-stranded DNA molecules. **b** The reverse transcription yield of different variants at different temperatures was determined by qPCR. Relative yields refer to the number of cDNA copies (assuming 100% PCR efficiency) produced by the WT RT. The values are based on at least three independent experiments. **c** Greedy cumulative path diagram at different temperatures. A line indicates an association between the variant and the parents, and the parents of each round have been marked in the figure

To evaluate the functionality of mutant proteins containing 11 hot-spot residues in various combinations, we utilized our previously established CFPS (Wu et al. 2022). This CFPS system provides an efficient platform with the unique capability to customize endogenous cellular functions, allowing for rapid and effective expression of proteins directly from in vitro linear DNA templates. To optimize MMLV RT production, we investigated the effect of varying the amount of polymerase chain reaction (PCR) product added to the standard 20 μL CFPS reaction. The results demonstrated that the protein yield initially increased with increasing template volume, reaching a peak of 42.9% (V/V) with 2 μL of PCR product before declining (Supplementary Fig. S1). Previous research has indicated that salt concentration and template stability significantly affect protein expression levels (Wang et al. 2018). Several strategies have been proposed to improve synthesis yields, including inhibiting native exonucleases (Sitaraman et al. 2004), increasing the template length (Hong et al. 2014), and employing DNA-binding proteins (Yim et al. 2020). To streamline the process, we used PCR amplicons as templates for the MMLV RT synthesis. A Twin-Strep-tag was fused to the C-terminus of MMLV RT to enable rapid purification using commercial streptavidin magnetic beads. The expressed protein was then quickly purified using commercial streptavidin magnetic beads. Following purification, the protein was incubated with a primer (Fig. 3a) and subjected to high-temperature conditions. Subsequently, the RNA template was introduced to initiate the reverse transcription process, with the efficiency of MMLV RT at specific temperatures directly correlated with cDNA production (see details in the Methods section).

Reverse transcription efficiencies were evaluated at various temperatures using a two-step reverse transcription PCR protocol, which comprised an initial cDNA synthesis at a constant temperature. Yields from the reverse transcription reactions, utilizing MS2 RNA as the template, were compared to those of the WT by qPCR at different temperatures (Fig. 3b). The results indicated that reverse transcription efficiency was enhanced in all MMLV RT variants with single mutant hot-spot residue, with the exception of the D86K and D502A, compared to WT at 37 °C. Among the variants, T284R exhibited the highest yields, with an efficiency that was 147-fold greater than that of WT. Meanwhile, the L413G, D502A, and D561N variants exhibited lower RT efficiencies than WT at 50 °C. Conversely, the T284R variant consistently demonstrated RT efficiencies four times higher than WT across all single mutations. Achieving enhanced protein properties often requires the introduction of multiple mutations during the engineering process. Beneficial mutations can be accumulated through directed evolution, where top mutations are fixed in each round of engineering (Zhang et al. 2022).

Theoretically, the combination of 11 hot-spot residues has a probability of 39.9 M, which is challenging to achieve through traditional screening methods. To save costs and quickly screen out the best variants, we sequentially combined only those mutant residues that demonstrated clear and substantial enhancement. This greedy approach may aid in efficiently identifying the optimal combination (Fig. 3c). In the initial selection process, the variants (D86K and D502A) were eliminated due to their inferior amplification efficiency relative to the WT at 37–50 °C. The T284R variant showed the highest yields of all variants at 37 °C and 55 °C and was used as the parent for the next round of screening. In the second round of screening, the amplification efficiency of M201 (E47K/T284R) and M202 (L413G/T284R) surpassed that of T284R, while all other mutants exhibited lower efficiency than T284R at 50 °C. This finding suggests that E47K and L413G exert a positive epistatic effect on T284R, whereas the others show a negative epistatic effect. Consequently, the E47K/T284R variant emerged as obviously enhanced when compared to other dual mutant variants. However, the amplification efficiency of M203 (T175A/T284R), M204 (H182R/T284R) and M205 (D561N/T284R) decreased significantly at 50 °C, resulting in discarding during subsequent screening. To quickly screen out the optimal variant, the M3 variant was constructed by binding to a variant with a higher amplification efficiency at 37–50 °C, which was then employed as the parent for subsequent screening. However, the M502 (E47K/T175A/E280R/T284R/D631V), M503 (E47K/E280R/T284R/D561N/D631V), and M504 (E47K/E280R/T284R/E585K/D631V) were designed using a production without greed combination strategy, which resulted in a lower amplification efficiency than the M5 variant. The results demonstrated that the greedy combination strategy, when employed in conjunction with mutations, facilitates a reduction in screening costs and the expeditious identification of local optimal solutions. Meanwhile, the greedy accumulation strategy has been successfully deployed in the highly efficient screening of biodegradable PETase enzymes (Cui et al. 2021). It is intriguing to note that, while the activity at 37 °C is considerably higher than at 50 °C for the same variant, the improvement at 37 °C lags significantly behind the activity at 55 °C during the greedy procedure following positive epistasis. This observation may be attributed to the fact that all hot-spot residues were primarily identified for enhancing the thermostability of RT. This finding aligns with prior research suggesting that combining more than two or three mutants does not necessarily lead to sustained improvements in enzyme performance (Chen et al. 2023).

### Enhanced reverse transcription activity and thermostability

**Fig. 4:**
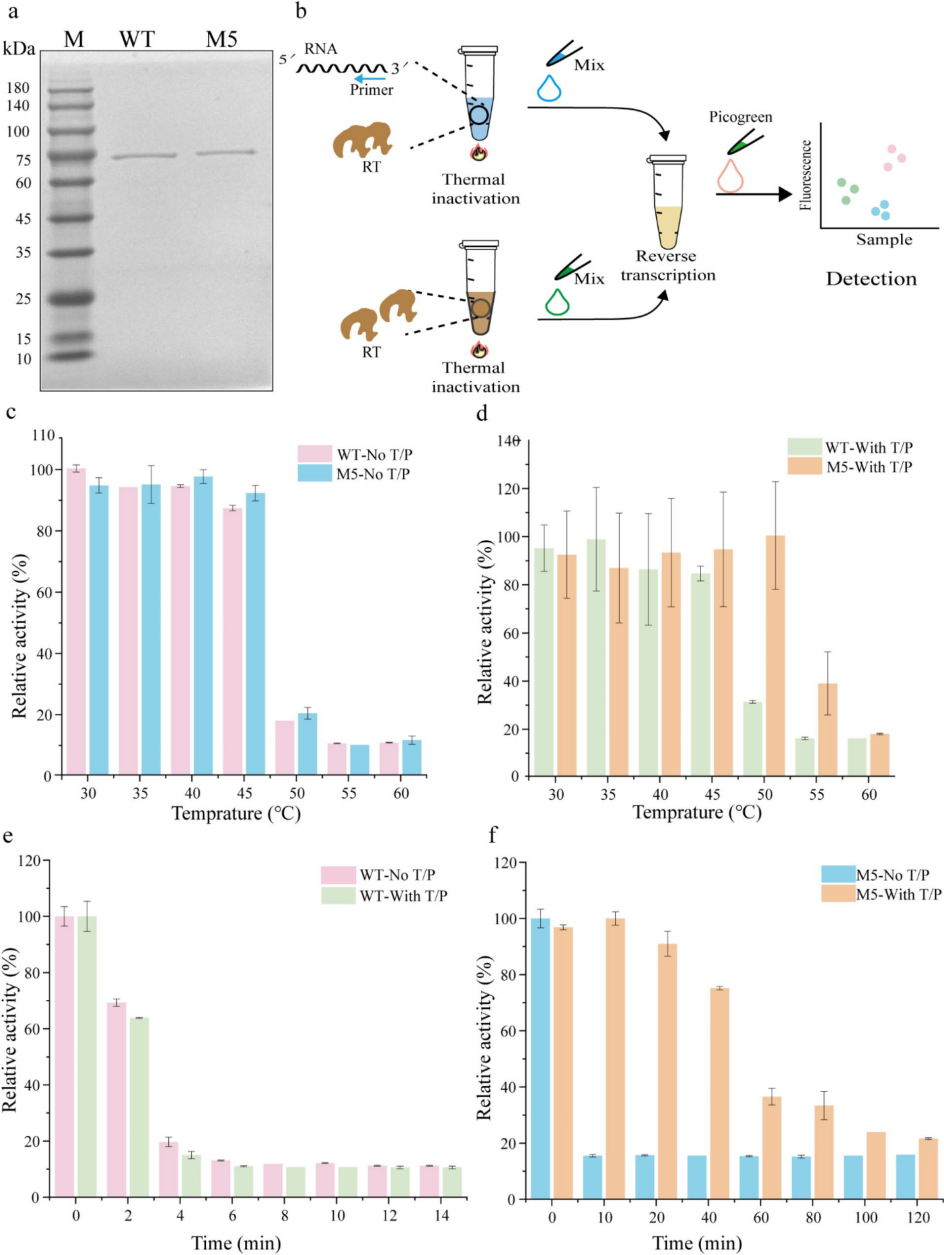
Determination of reverse transcription activity. **a** The SDS-PAGE of WT and M5 were carried out under reducing conditions. **b** Brief workflow for determining the kinetic stability of MMLV RT. The RTs was combined with or without primers, and the mixture was incubated at different temperatures. Subsequently, the fluorescence was quantified by introducing a dye, and finally, the enzyme activity was determined based on the established standard curve. **c**,** d** Thermal profiles. The relative activities were measured after 3 min incubations in the presence or absence of T/P at different temperatures. The percentage of activity was normalized relative to the maximum activity exhibited by each RTs (100%). **e**,** f** Half-lives. Half-lives were determined by incubating the RTs at 50 °C in the presence or absence of T/P for the times indicated

To achieve precise characterization of the mutant RTs, we purified the recombinant M5 protein with an N-terminal His_6_-tag in accordance with the standard procedure outlined in the Methods section. To assess the thermostability of the variant M5, we utilized the fluorescent dye PicoGreen to determine the optimal temperature and half-life of the enzyme, as determined by a standard curve (Supplementary Fig. S2). First, the WT and M5 proteins were expressed in BL21(DE3) cells and purified to homogeneity using immobilized metal affinity chromatography. The purity of M5 was confirmed via SDS-PAGE, with the molecular weights around 75 kDa (Fig. 4a). Next, the concentration of the M5 protein was measured using a standard curve (Supplementary Fig. S3). To evaluate the performance of M5 at elevated temperatures, both the protein with and without a template primer were subjected to thermal treatment and incubated at various temperatures (Fig. 4b). The optimum temperature is a critical characteristic of RTs. In the presence of a template primer, M5 demonstrated a broad range of thermal activity, maintaining over 80% activity between 30 °C and 50 °C. In contrast, the WT exhibited a narrower range of peak activity, with a maximum between 40 °C and 50 °C (Fig. 4c). In the absence of T/P, both M5 and WT displayed similar temperature ranges of 30 °C to 45 °C (Fig. 4d).

Next, we measured the enzyme half-life in the presence and absence of a template primer at 50 °C. M5 exhibited nearly complete activity under these conditions. In the presence of the template primer, the half-life of M5 exceeded 60 min, whereas that of the WT was less than 4 min (Fig. 4e-f). In the absence of the template primer, both M5 and WT showed minimal activity after 4 min at 50 °C. These results suggest that the M5 mutations improve thermostability, potentially through enhanced interaction forming a stronger interaction with the template primer, a conclusion that aligns with previous reports (Arezi and Hogrefe 2009).

**Fig. 5:**
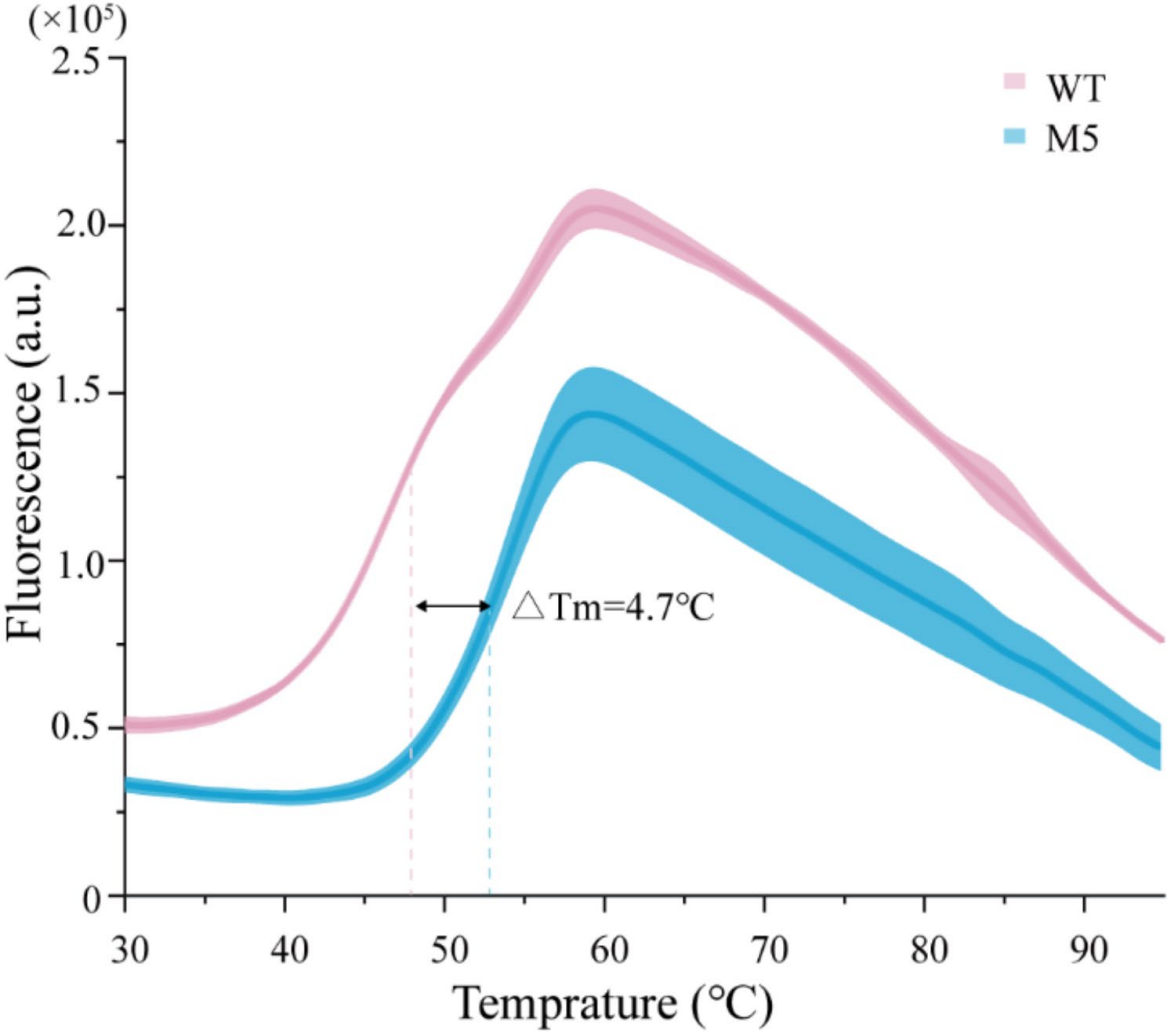
Determination of thermodynamics stability. PTSA curves of WT and M5 in the presence of T/P at a single elevated temperature (ranging from 30 °C to 99 °C)

To further assess the thermodynamic stability of the M5, we measured the Tm using SYPRO-ORANGE dye. This dye exhibits high fluorescence sensitivity and binds specifically to the hydrophobic patches of the protein that become exposed during unfolding. As the temperature increases, the protein unfolds, revealing hydrophobic residues and elevating the fluorescence signal. Our results demonstrated a 4.7 °C difference in Tm between M5 and the WT RTs in the absence of template primers (Fig. 5). This suggests that the M5 mutations enhance thermostability by improving the enzyme’s intrinsic stability. Notably, recent studies have documented increased intrinsic stability in MMLV RT through a combination of four mutant residues (E286R/E302K/L435R/D524A) (Yasukawa et al. 2010), which is distinctly different from the M5 mutations. This implies significant potential in combining multiple key residues to attain comparable thermostability improvements. In other words, epistasis offers substantial opportunities for protein engineering.

The aforementioned findings demonstrate that the M5 variant exhibits a broader temperature range, a longer half-life, and a higher Tm. To elucidate the impact of the M5 variant on the overall protein structure, AlphaFold was utilized to predict the structure of the WT. Subsequently, the structure of the M5 variant was predicted using Chimera based on the predicted structure of the WT. The MD simulations were performed on both the WT and M5 at 300 K, and their structural parameters were compared. The results demonstrated that the RMSD value of the M5 variant stabilized at approximately 2.2 Å after 20 ns. Whereas the RMSD of the WT stabilized at a higher value after 35 ns and remained higher than that of M5 beyond 50 ns (Supplementary Fig. S4a). This observation suggests that the M5 variant is more stable than the WT, consistent with the findings depicted in Fig. 5 above. To further investigate the effect of the mutation on the overall structure of the RTs, RMSF values were calculated for both the WT and M5. Comparatively, the RMSF of the M5 variant did not differ significantly from that of the WT in the range of Met1-Pro465. However, it was generally lower from Glu466-Leu649, indicating enhanced stability in the M5 variant relative to the WT (Supplementary Fig. S4b). Further analysis included the measurement of the Rg for both the WT and M5, in addition to RMSD and RMSF. The results demonstrated that the fluctuation trend of Rg of WT and M5 was similar. However, The Rg of the M5 was consistently lower than that of the WT, indicating increased compactness in the M5 variant (Supplementary Fig. S4c). To evaluate the structural impact of the mutation, the RMSD between the WT and M5 structures was calculated after 50 ns of MD simulation. The resulting RMSD value of 7.7 Å indicates significant structural divergence between the two variants (Supplementary Fig. S4d).

### Fidelity of MMLV RT variants

The fidelity of RTs refers to the sequence accuracy in the process of reverse transcription of RNA into DNA (Okano et al. 2018a). The fidelity of reverse transcriptase has been extensively applied in various fields, such as cDNA cloning, RNA sequencing, and disease diagnosis (Arezi and Hogrefe 2009). To assess the impact of mutations on RT fidelity, we evaluated the fidelity of the WT and M5 variants using next-generation sequencing (NGS). The results indicate that the error rate of these enzymes was 1.02 × 10^− 5^ (WT) and 1.20 × 10^− 5^ (M5) as shown in supplementary table S2. This is consistent with the reported fidelity of 6.30 × 10^− 5^ for the WT (Potapov et al. 2018). These findings suggest that introducing E47K/E280R/T284R/L413G/D631V (M5) mutation into WT does not impact fidelity.

## Discussion

**Fig. 6:**
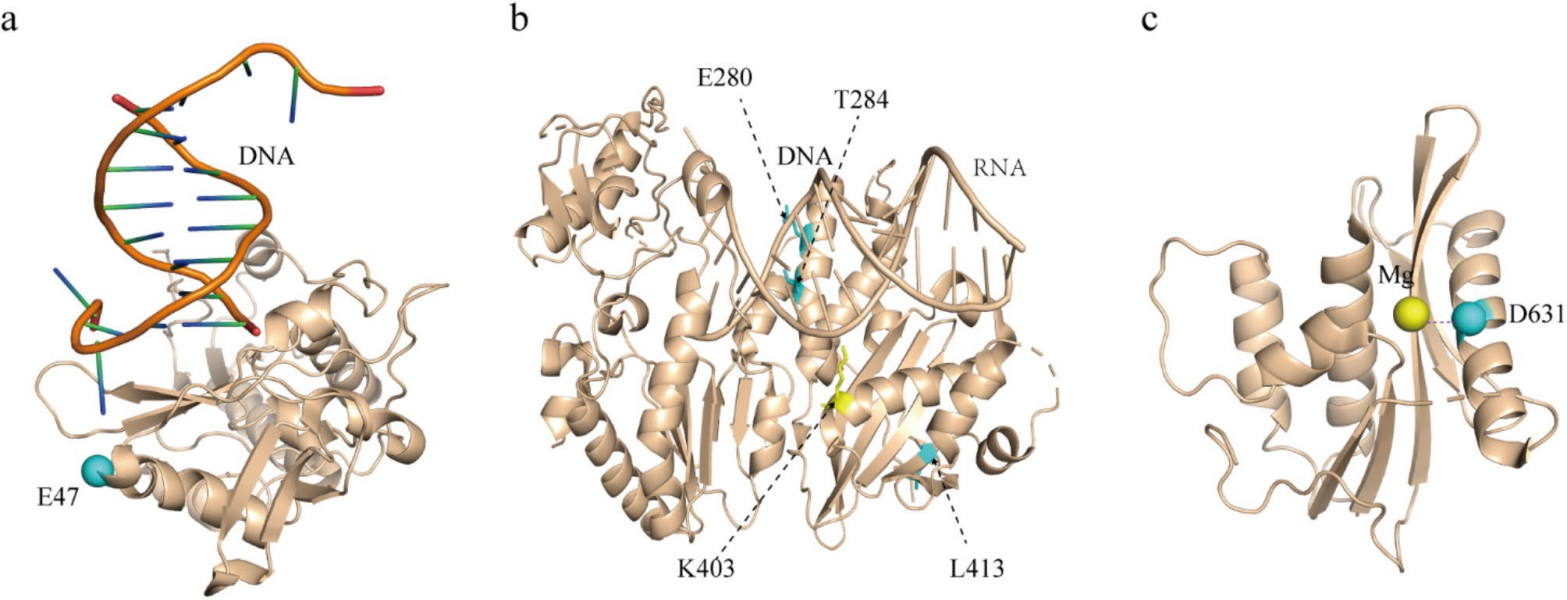
Structural context of thermostable mutations. **a** The amino acid residue E47, which interacted with the template primer, is visually represented as bronze spheres (PDB: 1D0E). **b** The cartoon representation of the crystal structure (PDB: 5DMQ) with E280 (cyan), T284 (cyan), L413 (cyan), and K403 (yellow) residues, which were highlighted as stick. **c** The D631 amino acid (bronze spheres) is located on the RNase H active site (PDB: 2HB5). Magnesium ions are colored in yellow

MMLV reverse transcriptase is of particular interest due to its high catalytic activity in reverse transcription. However, a significant challenge in cDNA synthesis reactions is the formation of secondary RNA structures, which can lead to truncated cDNA synthesis at lower temperatures. These secondary structures become destabilized at higher temperatures, but unfortunately, the WT exhibits poor thermal stability (Fig. 1b). In the context of protein evolution, the effects of individual mutations on the ability of MMLV RT to synthesize cDNA at elevated temperatures have been extensively studied. However, predicting the combined effects of mutation is complicated by the phenomenon of epistasis. For instance, while the L413G mutation alone has a detrimental effect on the protein’s thermal stability, it exhibits a beneficial effect when part of a combined mutation. This discrepancy may be attributed to epistatic interactions between the mutations. The L413 residue is located on the β-sheet at the connection domain. The L413 residue is a solvent-exposed residue, which forms a hydrophobic cluster together with the hydrophobic amino acids (410–414: LVILA). The L413K mutation showed increased solubility and close to WT (-78%) levels of polymerase activity, indicating that this position is variable but not essential for function. The mutation of L413 residue to a less hydrophobic G amino acid may have an effect on the hydrophobicity of the hydrophobic cluster, which may explain why the single-point mutation L413G is harmful to thermostability at 50 °C. The distance between the K403 residue and T/P is only 2.9 Å, which allows for the formation of polar interactions with small slot groups or phosphate backbone atoms (Nowak et al. 2013). The L413 residue is connected to the K403 residue by a short random coil (Fig. 6b). The L413G mutations may result in structural rearrangements that bind K403 more tightly to T/P. In addition, T284R and K403 are located in the same sequence region of T/P. The structural rearrangement caused by the L413G mutation increased the T/P binding capacity of the T284R and K403 mutations. Thus, in combination with mutations, L413G mutations show beneficial functions.

The thermal stability of the M5 variant is attributed to five mutant residues: E47K, E280R, T284R, L413G, and D631V, which are located in the fingers, thumb, connection, and RNase H domains, respectively. Notably, three of these mutations (E47K, E280R, T284R) are situated at positions previously implicated in interactions with the T/P (Fig. 6a-b) (Najmudin et al. 2000; Nowak et al. 2013). In a sequence-based alignment, MMLV RT T284 was identified as a counterpart to XMLV RT T306, which resides on the α-helix facing the minor groove of the T/P and interacts with the primer strand sugar-phosphate backbone (Nowak et al. 2013). The T284 residue is only 4.3 Å away from the T/P. The introduction of a positively charged T284R mutation may improve the binding of the T/P and enhance heat resistance by increasing the number of van der waals interactions formed by small slot groups or phosphate backbone atoms (Arezi and Hogrefe 2009), due to the negative charge of T/P. This likely explains why the M5 variant demonstrates enhanced thermal stability in the presence of the template primer. RTs possess two distinct catalytic sites: one for DNA polymerization and the other for RNase H activity (Menéndez-Arias et al. 2017). The RNase H activity of MMLV RT is crucial for cleaving RNA in RNA/DNA hybrids, a process that affects the efficiency of synthesizing long cDNA in vitro (Arezi and Hogrefe 2009). The active site of the MMLV RNase H enzyme includes a highly conserved motif, D-E-D (D524-E562-D583), along with a fourth conserved aspartate (D653), which are essential for RNase H activity (Lim et al. 2006; Skirgaila et al. 2013). Structural analyses reveal that these residues coordinate a magnesium ion within the catalytic core (Fig. 6c). The D631V mutation is known to inhibit RNase H activity (Lim et al. 2006). Interestingly, this mutation, along with the other mutants, contributes to increased thermostability in the presence of the template primer (Gerard et al. 2002).

## Conclusions

In this study, we investigated the thermostability of MMLV reverse transcriptase by combining mutations that increase surface charge and eliminate RNase H activity. From these combinatorial experiments, we identified the M5 variant, which exhibited an expanded thermal profile ranging from 30 °C to 50 °C. Additionally, M5 demonstrated a significantly prolonged half-life at 50 °C, increasing from less than 4 min to approximately 60 min in the presence of T/P. Additionally, M5 showed a 4.7 °C increase in Tm with the T/P. These findings indicate that the M5 variant has the potential to enhance the efficiency of biological reactions at elevated temperatures, thereby providing robust and reliable support for precision medicine and pharmaceutical development.

## Electronic supplementary material

Below is the link to the electronic supplementary material.

Supplementary Material 1

Supplementary Material 2

## Data Availability

All data and materials are available in the manuscript and supporting information.

## Abbreviations

CFPS: Cell-free protein synthesis
Ct: Threshold cycle
LETs: Linear DNA templates
MMLV RT: Moloney Murine Leukemia Virus reverse transcriptase
PCR: Polymerase chain reaction
PTSA: Protein thermal shift assay
RTs: Reverse transcriptase
T/P: Template-primer
Tm: Melting temperature
