# Supplementary figures and images for: Enhancing thermostability of Moloney murine leukemia virus reverse transcriptase through greedy combination of multiple mutant residues
Source: Bioresour Bioprocess. 2025 Feb 20;12(1):12. doi: 10.1186/s40643-025-00845-0 (PMC11842686; doi:10.1186/s40643-025-00845-0)

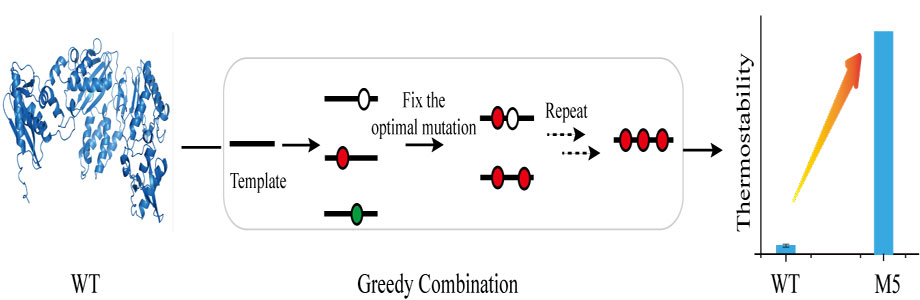

Supplement: Supplementary file 1 — Supplementary Material 1 [file 40643_2025_845_MOESM1_ESM.jpg]
