# Supplementary material for: Enhancing thermostability of Moloney murine leukemia virus reverse transcriptase through greedy combination of multiple mutant residues
Source: Bioresour Bioprocess. 2025 Feb 20;12(1):12. doi: 10.1186/s40643-025-00845-0 (PMC11842686; doi:10.1186/s40643-025-00845-0)
Supplement: Supplementary file 2 — Supplementary Material 2 [file 40643_2025_845_MOESM2_ESM.docx]

**Supporting Information**

Enhancing Thermostability of Moloney Murine Leukemia Virus Reverse Transcriptase through Greedy Combination of Multiple Mutant Residues

Youhui Yang^1,2^, Jie Zhang^1,2^, Zhong Li^1,2^, Hao Qi^1,2*^

^1^ School of Chemical Engineering and Technology, Tianjin University, Tianjin, China

^2^ Key Laboratory of Systems Bioengineering (Ministry of Education), Tianjin University, Tianjin, China

*** Correspondence:**Hao Qi
[haoq@tju.edu.cn](mailto:haoq@tju.edu.cn)

Table S1. DNA oligonucleotides used are listed.

| Oligo name | Sequence (5′-3′) |
| --- | --- |
| UR1 | CCAATCCGGATATAGTTCCTCCTTTC |
| URB1 | Bition-CCAATCCGGATATAGTTCCTCCTTTC |
| UFB1 | Bition-ATAGGGCTAGCAATAATTTTGTTTAACTTTAAG |
| UF2 | GATCTCGATCCCGCGGCGCTAATACGACTCACTATAGGGCTAGCAATAATTTTGTTTAAC |
| MSR-R4 | CCTCTAGCACACGGGGTGCAAT |
| G-F | GTCGCGGTAATTGGCGC |
| G-R | GGCCACGTGTTTTGATCGA |
| E47K-F | GTCTCAGAAAGCTCGTCTGGGTATCAAACCGCAC |
| E47K-R | CCCAGACGAGCTTTCTGAGACATCGGGTACTGTTTG |
| D86R-F | GTACCAACCGTTACCGTCCGGTTCAGGACCTGCGTG |
| D86R-R | CTGAACCGGACGGTAACGGTTGGTACCCGGTTTTTTAAC |
| T175A-F | CTCTCCGGCTCTGTTCGACGAAGCTCTGCACCG |
| T175A-R | GCTTCGTCGAACAGAGCCGGAGAGTTTTTGAAACCCTG |
| H182R-F | CTCTGCGTCGTGACCTGGCTGACTTCCGTATC |
| H182R-R | GTCAGCCAGGTCACGACGCAGAGCTTCGTCGAACAGGGTCGG |
| E280R-F | GCGTCGTTTCCTGGGTACCGCTGGTTTCTG |
| E280R-R | CAGCGGTACCCAGGAAACGACGCAGCTGACGCGGGGTTTTCGGG |
| T284R-F | CCTGGGTCGTGCTGGTTTCTGCCGTCTGTGGATC |
| T284R-R | GGCAGAAACCAGCACGACCCAGGAATTCACGCAGCTGACGCGGG |
| L413G-F | GGTTATCGGTGCTCCGCACGCTGTTGAAGCTCTGGTTAAAC |
| L413G-R | CAGCGTGCGGAGCACCGATAACCAGCGGCTGACCCATGG |
| D502A-F | GTACACCGCTGGTTCTTCTCTGCTGCAGGAAGG |
| D502A-R | GCAGAGAAGAACCAGCGGTGTACCAGGTGTGGTCAGCGTCCG |
| D561N-F | GTTTACACCAACTCTCGTTACGCTTTCGCTACCGC |
| D561N-R | GCGTAACGAGAGTTGGTGTAAACGTTCAGTTTTTTACCTTC |
| E585K-F | GACCTCTAAAGGTAAAGAAATCAAAAACAAAGAC |
| E585K-R | GATTTCTTTACCTTTAGAGGTCAGCAGACCACGACGACGG |
| D631V-F | GTATGGCTGTTCAGGCTGCTCGTAAAGCTGCTATCACCG |
| D631V-R | CGAGCAGCCTGAACAGCCATACGGTTACCACGAGCTTCAGCAG |
| E280R-F1 | GCGTCGTTTCCTGGGTCGTGCTGGTTTCTG |
| E280R-R1 | CAGCACGACCCAGGAAACGACGCAGCTGACGCGGGGTTTTCGGG |
| MSQ-F3 | ATCTCAGCCATGCATCGAGG |
| MSQ-R3 | ACTCCGGTTGAGGGCTCTAT |

Table S2. Analysis of fidelity of MMLV RT variants.

| Enzymes | M5 | WT |
| --- | --- | --- |
| Number of total reads | 2.96 × 10^6^ | 3.15 × 10^6^ |
| Number of reads with correct barcodes | 2.16 × 10^6^ | 2.23 × 10^6^ |
| Total base (A) | 3.06 × 10^8^ | 3.17 × 10^8^ |
| Number of errors (B) | 3,930 | 3,498 |
| Error rates (B/A) | 1.20 × 10^−5^ | 1.02 × 10^−5^ |


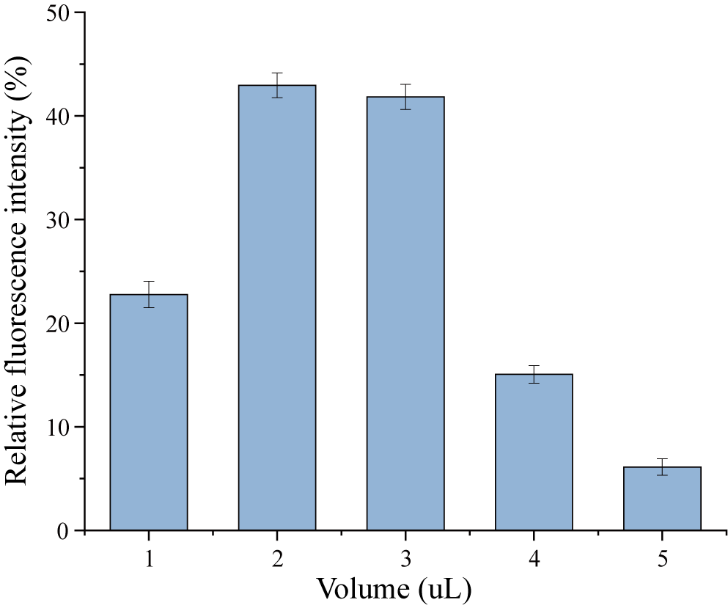


**Fig. S1** The optimized volume for the additive PCR amplicons in CFPS system.


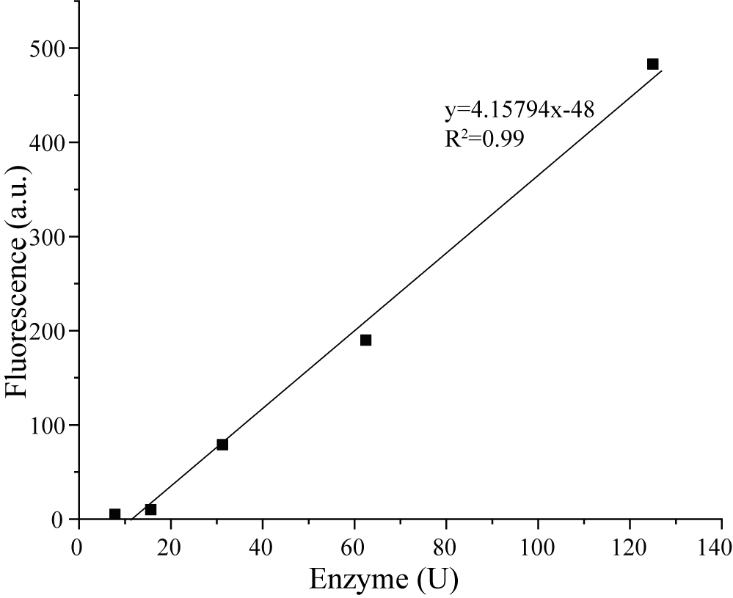


**Fig. S2** The standard curve between enzyme units and fluorescence-based on PicoGreen dyes.


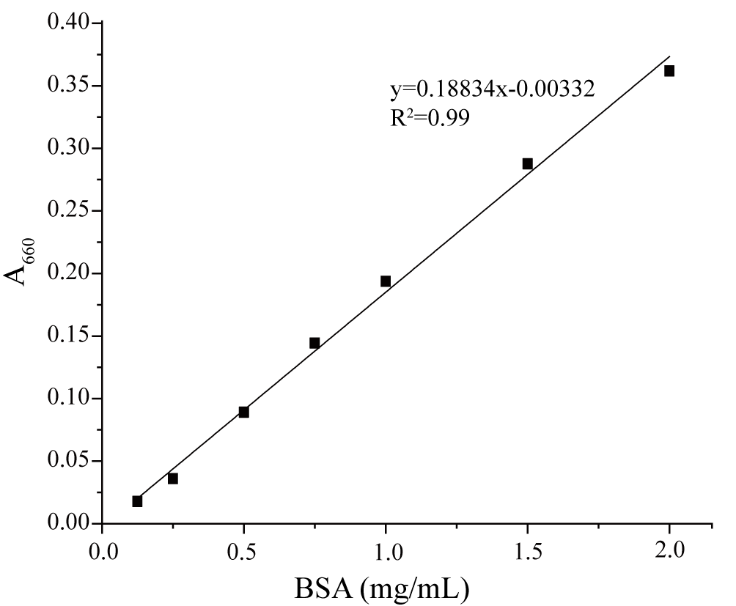


**Fig. S3** The standard quantitative curve of BSA based on the BSA protein concentration.


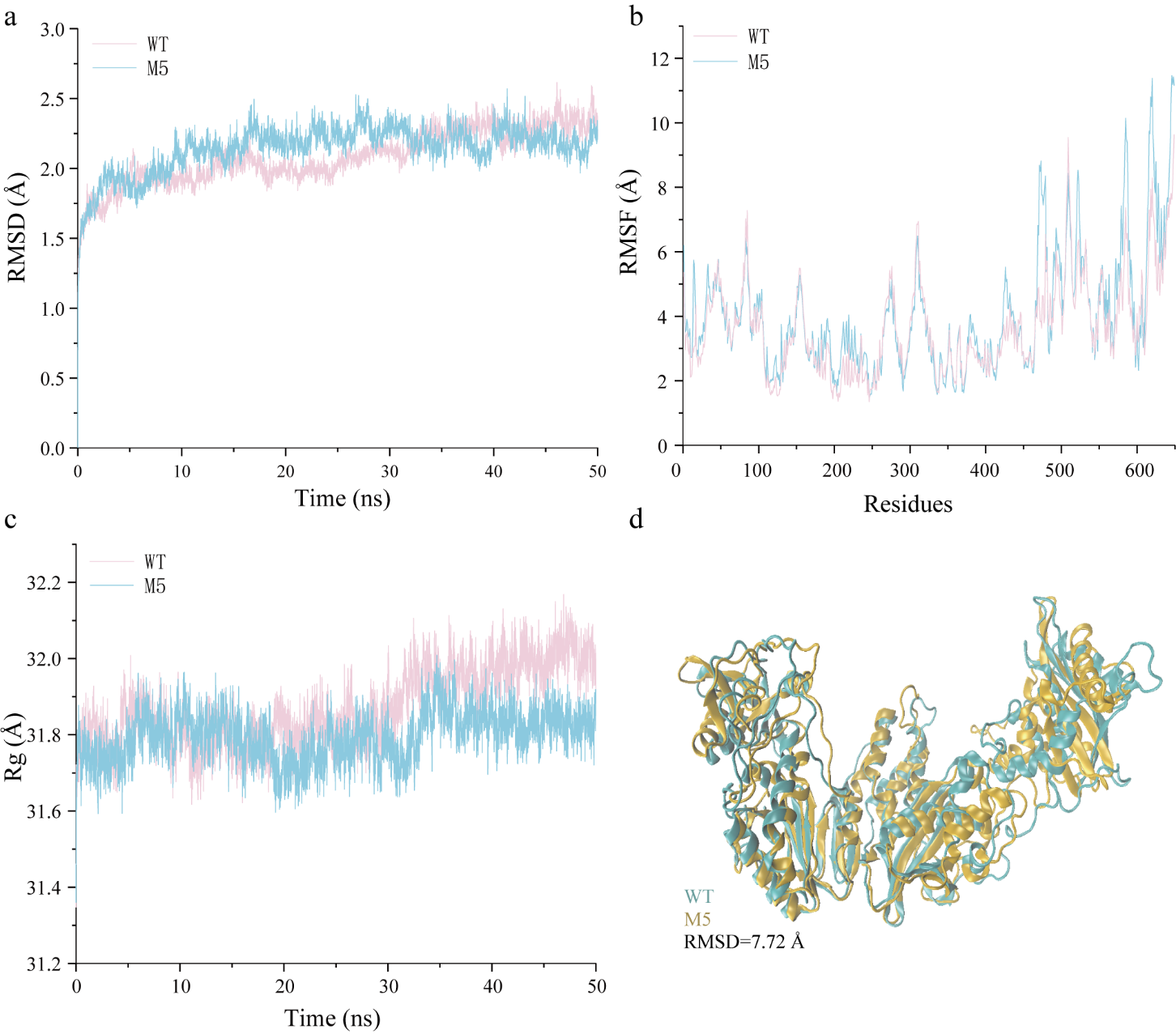


**Fig. S4** MD simulation comparison of WT and M5 variant at 300K. **a** RMSD. **b** RMSF. **c** Rg. **d** the RMSD of the WT and M5 was calculated based on the structure of the WT and M5 after MD simulation was carried out for 50 ns.
